# Niacin improves maturation and cryo-tolerance of bovine in vitro matured oocytes: An experimental study

**DOI:** 10.18502/ijrm.v17i9.5096

**Published:** 2019-09-22

**Authors:** Mojtaba Kafi, Mahboobeh Ashrafi, Mehdi Azari, Borhan Jandarroodi, Beheshteh Abouhamzeh, Arash Rakhshi Asl

**Affiliations:** ^1^ Department of Animal Reproduction, School of Veterinary Medicine, Shiraz University Shiraz Iran.; ^2^ Department of Basic Sciences, School of Veterinary Medicine, Shiraz University Shiraz Iran.; ^3^ Department of Anatomical Sciences, School of Medicine, AJA University of Medical Sciences Tehran Iran.

**Keywords:** Bovine, Embryonic development, Niacin, Oocytes, Vitrification

## Abstract

**Background:**

Nicotinic acid (niacin) is a broad-spectrum lipid-modifying agent that has potent antioxidant properties and reduces the production of lipid peroxidation.

**Objective:**

The purpose of the present study was to investigate the maturation, embryo development and cryo-tolerance merit, and levels of malondialdehyde (MDA), total oxidant status, and total antioxidant capacity following the supplementation of bovine oocytes maturation medium with different concentrations of niacin.

**Materials and Methods:**

Immature cumulus-oocyte complexes were cultured in tissue culture medium-199 maturation media supplemented with 0, 100, 200, and 400 *µ*M niacin under a standard in vitro culture condition. After 24 hr of culture, the nuclear maturation rate was assessed. Then, two groups of immature cumulus-oocyte complexes were cultured in TCM-199 either with or without 400 *µ*M niacin and evaluated for embryo development. Also, matured cumulus-oocyte complexes in both groups were frozen using a standard vitrification procedure. After vitrification, oocytes were warmed in two steps and evaluated for embryo development. In addition, the level of total antioxidant capacity, total oxidant status, and MDA were measured.

**Results:**

The results indicated that although the treatment with 400 *µ*M niacin increased in vitro nuclear maturation (87.6±5.3), it did not improved the embryo development to the blastocyst stage. Higher cleavage and blastocyst rates were observed in vitrified oocytes that were cultured with supplemented 400 *µ*M niacin compared to the control group (without niacin) (53.6±2.7 and 10.6±1.6 vs. 46.2±4.1 and 6.3±2.4, respectively). Also, the addition of 400 *μ*M niacin to the maturation media could decrease MDA levels after maturation.

**Conclusion:**

Niacin could improve the quality of in vitro embryo production (IVP) embryos and tolerance of bovine oocytes to vitrification.

## 1 Introduction

In recent decades, successful reproductive technologies have been developed in farm animals. One of the benefits of in vitro embryo production (IVP) is generating large numbers of embryos at low costs. However, the IVP embryos have lower quality than those produced in vivo. Therefore, to optimize IVP outcome acquiring the necessary factors for bovine oocyte metabolism is essential. The first and most critical step in the achievement of IVP is maturation of oocyte in vitro. Culture condition is one of the major factors influencing the efficiency of IVM. Under the in vitro atmospheric oxygen tension (20% O_2_), a high concentration of reactive oxygen species (ROS) is generated that could joint to cellular macromolecules such as lipids and promote lipid peroxidation (LPO), resulting in cell damage (1). Therefore, reducing ROS in germ cells by antioxidants could improve their developmental merit. The deficiency of antioxidant capacity within follicle has shown to decrease the maturation competency of the oocyte and, therefore, interfere in the process of fertilization and development (2,3). Enzymatic antioxidants such as catalase (CAT), superoxide dismutase (SOD), and the level of non-enzymatic antioxidants including L-carnitine, cysteamine, cysteine, and N-acetyl-L-cysteine, have been used to solve this problem (4,5).`` Nicotinic acid (niacin) is a lipid-modifying agent that decreases plasma triglyceride and low-density lipoprotein while raising high-density lipoproteins" (6). Also, pieces of evidence from studies supported that Nicotinamide (NA) has potent antioxidant properties and its deficiency increases oxidative stress (7,8). These studies showed that niacin status could affect the stir of several antioxidant enzymes such as SOD, CAT, and Glutathione Reductase. Furthermore, niacin could reduce the generation of LPO biomarkers such as malondialdehyde (MDA) in rats (8,9). Also, it has been shown that the addition of niacin to embryo culture in the condition of heat shock could relatively improve embryo development to the blastocyst stage (10).

To our knowledge, no study has reported the effect of niacin supplementation to IVM medium on nuclear maturation, developmental competence, and cryo-tolerance in bovine oocytes. We hypothesized that niacin could improve the quality of IVP embryos and cryo-tolerance of bovine oocytes, either by antioxidant activity or by reducing LPO. Therefore, the present study was designed to evaluate the effects of niacin addition during IVM on nuclear maturation and cryo-tolerance after vitrification in the bovine oocyte. In addition, the level of total antioxidant capacity (TAC), total oxidant status (TOS), and MDA were measured.

## 2 Materials and Methods

All chemicals were purchased from Sigma (St. Louis, MO, USA) unless otherwise indicated.

### 2.1 Experimental design

This experimental study was carried out in the IVF Laboratory at the School of Veterinary Medicine, Shiraz, Iran, between September and October 2017.

Experiment 1: In vitro maturation of bovine oocytes was performed in the presence of niacin at different concentrations (0, 100, 200, and 400 *µ*M). At the end of maturation, cumulus cell expansion and nuclear maturation rates were determined.

Experiment 2: Based on the results of experiment 1, 400 *µ*M concentration were used to investigate the effects of niacin supplementation in IVM medium on their subsequent developmental competence and cryopreservation. In vitro matured oocytes in the presence of niacin (0 and 400 *µ*M) were vitrified. Next, they were thawed after a week. Vitrified and nonvitrified oocytes were fertilized and then cultured for eight days. Cleavage, morula, and blastocyst rates were determined on days 2, 6, and 8 of in vitro culture, respectively. For further investigation, the levels of TAC, TOS, and MDA were measured in immature oocytes, which were matured in 400-µM niacin and matured without niacin (control group).

### 2.2 Oocyte recovery

Cattle ovaries were collected from the Shiraz abattoir and transported to the laboratory in normal saline solution at 35ºC, within 3 hr. The cumulus-oocyte complexes (COCs) were recovered from healthy follicles (2–8 mm follicles) with a 20G needle. Excellent and good quality oocytes were selected for IVM (11).

## Experiment 1: Effects of niacin addition in IVM medium on oocyte nuclear maturation

To determine the effects of niacin on nuclear maturation, a total of 676 good or excellent quality COCs were randomly assigned to four groups: control (CG, n= 142) without niacin, treated with niacin 100μM (N100, n = 157), niacin 200μM (N200, n= 188), and niacin 400μM (N400, n =189). In all the selected experimental groups, the COCs were washed in washing medium (HEPES-buffered TCM-199 supplemented with 10% fetal calf serum (FCS)) and cultured in IVM medium (TCM-199 supplemented with 10% heat-treated FCS), and with 0.1 IU/mL recombinant human FSH (Follitrope, LG Life Sciences, South Korea), 5 IU/mL highly purified hCG (Karma, Pharmatech GmbH, Germany), and 50 *μ*g/ml gentamicin supplemented with niacin at 0, 100, 200, and 400 *µ*M (control, N100, N200 and N400 groups, respectively). Groups of 30–40 COCs were incubated in 500 *μ*l equilibrated maturation medium in four-well culture dishes (Nunc™, Denmark) for 24 hr at 38.5ºC in 5% CO_2_ at 90% humidified atmosphere. The experiment was performed in five replicates (12).

### 2.3 Evaluation of nuclear maturation of oocytes

After maturation, the degree of cumulus expansion was scored under a stereo zoom microscope based on a subjective scale of 0 to 2, where 0 indicates no detectable expansion and 2 indicates full expansion. To evaluate meiotic progression, the oocytes were denuded by frequent pipetting, mounted on glass microscope slides under coverslips and fixed for at least 24 hr with acetic alcohol (1:3) and then stained with1% aceto-orcein (1% orcein in 45% glacial acetic acid) and examined for nuclear morphology with a compound microscope at ×100 and ×400 magnifications. "Oocytes were classified as follows: immature (did not reach metaphase), mature (presented a metaphase II plate and the polar body), and abnormal (any chromosomal aberrations such as diploid, abnormal metaphase II, multidirectional spindle, and chromosomal dispersion)" (13).

## Experiment 2: Effects of niacin addition in IVM medium on cryotolerance and subsequent embryonic development

To evaluate the effects of niacin on cleavage rate and embryo development, a total of 677 oocytes were divided into four groups: (1) culture in IVM medium that was not vitrified (CG, n= 182); (2) culture in IVM medium with 400 *µ*M niacin that was not vitrified (CN, n= 177); (3) culture in IVM medium that was vitrified (CV, n= 158); and (4) culture in IVM medium with 400 *µ*M niacin that was vitrified (NV, n=160). Following maturation, oocytes were transferred into 500µl of fertilization medium (modified Tyrode’s medium) in four-well dishes (Nunc™, Denmark) (50 COCs/well). Frozen semen that was previously tested in the lab was used for fertilization. Motile spermatozoa were acquired by the swim-up method and were added to wells containing oocytes at a final concentration of 106 (10 to power 6) spermatozoa ml−1 (ml to power −1). Oocytes and spermatozoa were incubated together for 24 hr at 38.5ºC in 5% CO_2_ under maximum relative humidity. At the end of the fertilization period, presumptive zygotes were completely denuded by repeated pipetting and transferred into the culture medium (modified synthetic oviduct fluid (mSOF) supplemented with 4 mg/ml fatty acid-free BSA) and cultured in four-well dishes at 38.5ºC for eight days of 5% CO_2_ under maximum relative humidity. The embryo culture medium was renewed every 48 hr. Fertilization day was considered as day 0. Embryonic cleavage, morula, and blastocyst rates were determined on days 2, 6, and 8 of in vitro culture, respectively (13,14). This experiment was performed in five separate replicates.

### 2.4 Vitrification and warming of matured oocytes

After IVM, COCs were partially denuded in the presence of 1 mg/ml hyaluronidase for 1min. A group of 3–5 oocytes was placed in equilibration solution (7.5% dimethylsulfoxide (DMSO) and 7.5% ethylene glycol (EG) in holding medium (HM; TCM-199-HEPES+20% FBS)) for 9 min and then transferred to vitrification solution (15% DMSO, 15% EG, and 0.5 mol/L sucrose in HM) for 45 sec at 25ºC. Oocytes were loaded onto the tip of Cryotop (Kitazato Supply Co., Tokyo, Japan) in a small volume of vitrification solution and plugged into liquid nitrogen immediately. Warming of vitrified oocytes was done by immersing the Cryotop tip directly in a 37ºC warming solution composed of HM and 1 M sucrose for 1 min, followed by treatment with HM supplemented with 0.5 M for 3 min (15). Afterward, the retrieved oocytes were washed and transferred to HM until the next procedure.

### 2.5 Measurement of MDA, TAC, and TOS in oocytes

To determine the effects of niacin on the levels of MDA, TAC,and TOS following maturation, 50 expanded COCs were collected from each experimental group (i.e., immature oocytes, control mature oocytes without niacin, and oocytes matured with niacin) and washed three times in phosphate-buffered saline (PBS). A number of 50 oocytes in 50 *μ*l of PBS were frozen at -20ºCuntil use. After repeated freeze-thaw cycles for lysis of cells, the levels of mentioned parameters were measured in all groups with specific kits (ZellBio GmbH, Germany) according to the manufacturer’s instructions.

### 2.6 Ethical consideration

This study was approved by the ethical working with animals and research committee of the School of the Veterinary Medicine, Shiraz University (94GCU6M1251).

### 2.7 Statistical analysis

One-way analysis of variance (ANOVA) and post Tukey test were used for comparing expansion of cumulus cells, oocyte maturation, embryo development (cleavage and blastocyst rate), and the levels of MDA, TOS, and TAC among experimental groups (significance at p< 0.05). All statistical analyses were performed using a computer-aided statistical software package (IBM®SPSS Statistics version 22 for windows). Also, all data are presented as mean±SD.

## 3 Results

### 3.1 Effects of niacin addition in IVM medium on nuclear maturation

After in vitro oocyte maturation, no difference was observed in the mean percentage of partially and fully expanded COCs among groups (Table I). In the present study, the supplementation of IVM medium with niacin improved nuclear maturation of oocytes (Table II). The highest nuclear maturation rates were acquired in the N400 group (87.6 ± 5.3%) which was significantly higher than that in the control group (78.1 ± 3.7), N100 (75.7 ± 5.1) and N200 (76.5 ± 5.2) (p = 0.03, p < 0.001, and p = 0.01, respectively). No significant difference was observed in nuclear maturation between other groups (Table II).

**Table I:** Mean (±SD) percentages of cumulus cell expansion following the addition of niacin at different concentration to the maturation media

Group	N	Grade 2	Grade 1	Grade 0
Control	152	114 (76.06 ± 6.9)	33 (21.03 ± 5.8)	6 (3.3 ± 3.2)
N100	120	90 (75.4 ± 4.8)	22 (18.6 ± 1.9)	8 (5.9 ± 5.1)
N200	117	82 (71.5 ± 8.1)	34 (27.3 ± 14.8)	10 (8.09 ± 1.7)
N400	190	143 (72.4 ± 15.1)	34 (20.3 ± 11.9)	13 (7.2 ± 4.9)

Data presented as n (%)

Grade 2, full expansion; Grade 1, partial expansion; Grade 0, no expansion

**Table II:** Mean (±SD) percentages of nuclear maturation following the addition of niacin to the maturation media

Group	N	Mature	Immature	Abnormal
Control	142	110 (78.1 ± 3.7)	22 (16.2 ± 4.1)	10 (5.6 ± 6.5)
N100	157	119 (75.7 ± 5.0)	26 (15.5 ± 7.4)	12 (8.7 ± 8.1)
N200	188	144 (76.5 ± 5.2)	27 (13.9 ± 4.4)	17 (9.6 ± 5.9)
N400	189	167 (87.6 ± 5.3)^*^	8 (4.3 ± 1.7)^*^	14 (7.9 ± 5.5)

Data presented as n (%)

The N100, N200 and N400 have treated groups with 100, 200, and 400μM of niacin, respectively

* Represents a significant difference between N400 and other groups at P<0.05 level; data were compared by One-Way Analysis of Variance (ANOVA) and post Tukey’s test

### 3.2 Effects of niacin addition in IVM medium on embryonic developmental competence of non-vitrified and vitrified IVM oocytes

Following IVF and IVC, no difference was observed in cleavage and embryo development between non-vitrified control and N400 groups (Table III). As shown in Table IV, in the vitrified groups, the cleavage rate, blastocyst formation, and cleaved/blast rate were significantly higher in the niacin vitrified (NV) group than the control vitrified (CV) group (p< 0.001) (53.6±2.7, 10.6±1.6 and 19.9±3.2 vs. 46.2±4.1, 6.3±2.4 and 13.6±4.4, respectively).

**Table III:** Mean (±SD) percentages of cleavage, morula, and blastocyst yield following in vitro maturation of oocytes in addition of 400 *μ*M niacin

Group	N	Cleaved^*^	Morula^*^	Blastocyst^*^	Blast/cleaved^**^
CG	182	137 (75.3 ± 1.1)	74 (40.6 ± 3.4)	39 (21.5 ± 1.4)	28.6 ± 1.8
CN	177	135 (76.3 ± 0.8)	74 (41.7 ± 3.3)	36 (20.2 ± 2.4)	26.5 ± 3.2

* Data presented as n (%);

** Data presented as %

CG, culture in IVM medium and not vitrified; CN, culture in IVM medium with 400 *µ*M niacin and not vitrified. Comparisons among groups were performed using One-Way ANOVA and no significant difference was observed between groups

**Table IV:** Mean (±SD) percentages of cleavage, morula, and blastocyst yield following vitrification of mature oocytes in addition to 400 *μ*M niacin

Group	N	Cleaved^**^	Morula^**^	Blastocyst^**^	Blast/cleaved^*^
CV	158	73 (46.2 ± 4.1)	37 (23.2 ± 3.4)	10 (6.3 ± 2.4)	13.6 ± 4.4
NV	160	86 (53.6 ± 2.7)^#^	41 (25.6 ± 2.4)	17 (10.6 ± 1.6)^#^	19.9 ± 3.2^#^

* Data presented as n (%); ^**^Data presented as %

CV: control vitrified oocytes group; NV: niacin vitrified oocytes group

^#^Represents a significant difference between CV and NV group at p< 0.001 level. Data were compared by One-Way Analysis of Variance (ANOVA) and post Tukey’s test

### 3.3 Effects of niacin supplementation in IVM medium on the levels of MDA, TAC, and TOS

**Figure 1:**
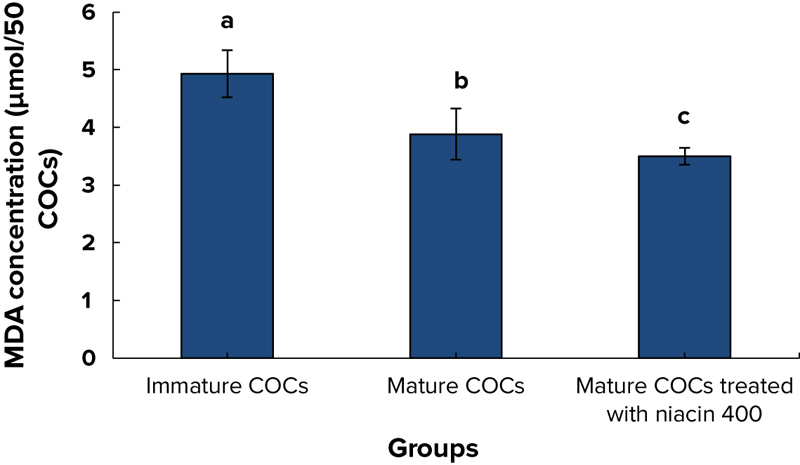
The level of MDA in all groups (mean±SD); a, b, and c represent a significant difference between groups at p < 0.05 level.

The level of MDA in the immature group was significantly higher than the control mature and niacin mature groups (p = 0.04 and p = 0.02, respectively). In addition, the level of MDA in niacin mature group was significantly lower than the other groups (Figure 1). No difference was observed in the levels of TAC (0.07, 0.08, and 0.08 mmol/50 for immature COCs, mature COCs, and mature COCs in niacin group, respectively) and TOS (6.8 ± 0.3, 6.7 ± 0.4, and 6.8 ± 0.3 *µ*Mol/50 for immature COCs, mature COCs, and mature COCs in niacin group, respectively) among experimental groups.

## 4 Discussion

This experimental study is the first to show that addition of niacin (400 *µ*M) could positively affect nuclear maturation and blastocyst yield of vitrified bovine oocytes. Nuclear maturation of oocytes is one of the critical steps in the success of IVP. The results showed that niacin treatment with 400 *µ*M concentration during IVM could increase nuclear maturation rates and improve the developmental ability to the blastocyst stage of vitrified oocytes. The oxygen concentration in culture (in vitro) is higher than that in an in vivo environment, leading to an increased level of ROS (1). Therefore, the antioxidant effect of niacin may be a principal factor for the improved development in the niacin-supplemented group. In addition, niacin is a lipid-modifying agent that decreases plasma triglyceride. It may reduce the intracellular lipid content in oocytes and, consequently, improve tolerance to cryo-damage. However, further investigations are needed to clarify the exact cellular processes of niacin treatment during oocyte maturation. Similar to niacin, treatment of IVM medium with L-carnitine with antioxidant activity has improved embryo developmental merit of IVM oocytes after freezing (16). Since vitrification increases ROS in vitro, oxidative stress causes damage to macromolecules, including lipids, proteins, carbohydrates, and DNA in all cells of mammals (17). High ROS concentrations have harmful effects on germ cells and embryos. Oxidation of unsaturated fatty acids is one of the most important harmful effects of LPO. MDA is one of the end stable products of LPO that can be used as a cumulative measure of LPO (18,19). MDA levels are also used as a marker in predicting the outcomes of assisted reproductive techniques (ART). It has been shown that there is a relationship between the levels of MDA in follicular fluid and cleavage rates in women treated in ARTs. The high levels of MDA indicated a negative correlation with fertilization rate and a negative influence on embryonic development competence (20,21). In the present study, the level of MDA was reduced significantly in niacin-treated group compared to the other groups. In several animal studies, it has been shown that niacin deficiency could adversely affect LPO and niacin administration could decrease the production of LPO biomarkers (8,9,22). It has been proved that TAC of ovarian follicular fluid has a positive relation with oocyte maturation rate and can serve as a predictive marker of human IVF success. Chattopadhayay and colleagues suggested that the TAC level in follicular fluid of immature oocytes would be lower compared to the mature oocyte in polycystic ovarian syndrome (PCOS) patients (23). Also, Oyawoye and colleagues have shown that a higher TAC level increases fertilization potential in women undergoing IVF (24). Several studies have indicated that niacin could increase the level of glutathione and the activity of several antioxidant enzymes such as SOD, CAT, and GPx (8,22).

Although in our study, the nuclear maturation of oocytes in niacin-treated group was increased significantly, the levels of TAC and TOS did not change. In comparison, the level of MDA significantly decreased in niacin-treated group compared to the other groups. In the study of Pasqualotto and colleagues, no association of TAC level, oocyte maturity, fertilization, and cleavage was reported with embryo quality (2). It seems that MDA is better than TAC as an oxidative stress marker, which has also been proven in the study of Agarwal and colleagues (25). In this regard, the niacin receptor was identified on adipose tissue by Tunaru and colleagues (26).

According to relevant reports, many of the beneficial and adverse effects of niacin are mediated via a G protein receptor, GPR109A (27,28). Also, the GPR109A receptor was detected in bovine oocytes and preimplantation embryos (10). In recent years, it has been reported that niacin can alter the expression of several genes in different tissues (29). The activation of GPR109A can inhibit the activity of adenylatecyclase that resulted in the reduction of cAMP. "The decrease in the level of cAMP in cells could affect the cAMP targets such as protein kinase A (PKA) and exchange protein activated by cAMP (Epac). It is known that PKA and Epac as two factors are involved in the expression of several genes" (30).

Therefore, in our study, the beneficial effects of niacin in maturation medium could be mediated also via the GPR109A receptor and the effect on gene expression; however, further investigations are needed to clarify this subject.

## 5 Conclusion

Overall, our findings support the hypothesis that niacin in 400 *µ*M concentration could improve the maturation and tolerance of bovine oocytes to vitrification by reducing LPO.

## Conflict of Interest

The authors declare that they have no conflict of interest.
